# Is Coping with Stigma by Association Role-Specific for Different Family Members? A Qualitative Study with Bipolar Disorder Patients’ Relatives

**DOI:** 10.1007/s10597-021-00809-6

**Published:** 2021-03-09

**Authors:** Hélène Richard-Lepouriel, Jean-Michel Aubry, Sophie Favre

**Affiliations:** 1 Mood Disorder Unit, Psychiatric Specialties Service, Geneva University Hospital, Rue de Lausanne 20, 1201 Geneva, Switzerland; 2 Department of Psychiatry, University of Geneva, Geneva, Switzerland

**Keywords:** Bipolar disorder, Stigma by association, Relatives, Qualitative study, Coping, Family roles

## Abstract

Trying to cope with stigma by association (SBA) often results in behaviors leading to social isolation and withdrawal. This study aimed at exploring the stigma-related experiences of family members of persons living with bipolar disorder (PW-BD). A semi-structured interview was conducted with relatives of PW-BD. Open-ended questions addressed three issues: awareness of public stigma of bipolar disorder, experiences of associative stigma, and ways of coping with experiences of SBA. Data were collected from a purposive sample of 21 family members. Experiences of SBA were specifically related to the different family roles. Parents had to deal with responsibility, partners with the choice of staying or not, and siblings with “a sort of duty.” These specific prejudices enhanced specific coping strategies. This is the first study to highlight specific issues and coping from the perspective of family members. Based on these findings, specific targeted interventions could be developed.

## Introduction

Public stigma (which refers to the discrimination and devaluation by others) of families having a member coping with mental health conditions has been described by Park and Park (2014) who reviewed the relevant literature to analyze the concept. They identified three key attributes of family stigma (others’ negative perceptions, attitudes, emotions, and avoidant behaviors toward a family (and every family member); others’ belief that the unusualness of the family is somehow harmful, dangerous, unhealthy, capable of affecting them negatively, or different from general social norms; others’ belief that the family members are directly or indirectly contaminated by the problematic family member). These public attributes have consequences, that can be emotional (e.g., fear, guilt, shame), social (e.g., discrimination, having a poor reputation), and interpersonal (e.g., avoid social relationships, social isolation), leading to a decrease in the family’s quality of life.

Perception of devaluation by the general public towards self and family impact both self-stigma in people living with a mental illness and stigma by association (SBA) in the family members. SBA refers to a broad construct including perceived stigma, experienced stigma and sometimes internalized stigma by relatives of people with mental illness. According to some authors, 75% of the family members of persons living with a mental illness perceived that they were being stigmatized (Shibre et al. 2001). The highest level of stigma by association was evident among caregivers of persons living with schizophrenia, followed by care providers of persons living with bipolar disorder (PW-BD) and depressive disorder (Chang et al. 2017; Grover et al. 2017). Gonzalez et al. (2007) interviewed 500 caregivers of patients participating in the Systematic Treatment Enhancement Program for Bipolar Disorder (STEP-BD) study. Two groups were formed based on the patients’ clinical status (unwell group versus well group). Stigma was prevalent among caregivers of PW-BD in both the groups, and was related to whether symptoms were exacerbated or remitted.

Research has shown that SBA can be a major source of psychological distress, such as depression, stress (van der Sanden et al. 2016; Shi et al. 2019), and diminished quality-of-life. Family members reported experiencing social isolation, shame, self-blame and fear of public reaction after their relative disclosed their mental health problem (Park and Park 2014; Nxumalo and Mchunu 2017; Huggett et al. 2018). In addition, 18% of the relatives had at times believed that the patient would be better off dead, and 10% had experienced suicidal thoughts (Östman and Kjellin 2002). SBA and associates’ response to stigma can potentially also impact diagnosed individuals who may be particularly sensitive to their relatives' reactions (Yanos 2018).

Family burden is not just a consequence of SBA, but also concerns how one copes with stigma (Yanos 2018). Studies explored the experience of stigma and coping strategies among relatives of PW-MI (van der Sanden et al. 2014, 2016; Nxumalo and Mchunu 2017; Bonsu and Salifu Yendork 2019; Baron et al. 2019). SBA was more likely to lead to distress when it was coped with in a “maladaptive” way (e.g. through self-blame, denial, or behavioral disengagement). Meanwhile, effective coping styles such as acceptance and social support reduced stress among family members. These results suggest that the management of PW-BD must also focus on stigma and psychological distress among relatives, and, accordingly, plan intervention strategies to reduce stigma. It is imperative to have a deeper understanding of these subjective processes in order to provide an advanced account, an assessment of SBA, and coping strategies among relatives of people living with mental illness. Qualitative investigations are optimally positioned as a first step in this endeavor.

The research question of this study was focused on SBA in bipolar disorder patients’ relatives. The aim of this qualitative research is (1) to explore the stigma-related experiences of family members of persons living with BD, (2) to elucidate how relatives perceive, experience and cope with SBA, and (3) to assess whether coping differs in accordance with their roles in the family.

## Methods

### Design and Setting

This qualitative study involved an in-depth interview that focused on associative stigma. Two investigators participated in the study—a psychiatrist and a psychologist. The interviews were semi-structured and sufficiently amenable to allow discussion. Open-ended questions focused on three issues: awareness of public stigma of BD, experiences of associative stigma and strategies to cope with experiences of SBA. There was no direct question on the interaction of families with the community, friends, and colleagues. These questions were developed following a review of the literature.

### Participants

Participants were recruited from the mood disorder unit of Geneva's University Hospitals (Switzerland). They were enrolled either directly (group for relatives of PW-BD or couple therapy) or indirectly (via PW-BD). No financial incentive was provided to the participants.

### Procedure

The semi-structured interviews were conducted by one of the investigators and were audio-recorded. The mean interview duration was 37.8 min (min: 27–max: 58; SD: 8.6). The participants were encouraged to narrate their experiences, representations and strategies related to SBA. Probes and reflective listening were used to elicit in-depth responses. Interviews were transcribed verbatim by the psychologist who conducted the interviews (n = 15) or an assistant (n = 6). The number of interviews was defined by the emergence of redundancy of topics, in accordance with the principle of data saturation (Saunders et al. 2018). After the 21st interview, thematic saturation was reached.

### Data Analysis

A mixed inductive and deductive approach using a step-by-step guide (Braun and Clarke 2006) was used for the thematic analysis of the transcripts. Both investigators read the transcripts and coded them independently. Subsequently, they compared and contrasted their coding during five sessions. They made a consensus on the themes that were representative of the codes and related to SBA. The code and themes proved accurate when re-tested.

## Results

### Participants

The sample consisted of 21 relatives who were recruited from relatives' psychoeducational groups (48%), via PW-BD (43%), and through couple therapy (9%). The sample primarily constituted women (67%), mean age was 53 (min: 28–max: 62; SD: 13.7), and the relatives were mainly from Western Europe (76%). The relatives in this sample had secondary level education or above, 62% had a university degree, 29% a college diploma and 9% some other diploma. Most relatives were professionals (28%), some were managers (19%), some technicians (14%), some retired (24%), some unemployed (10%) and one (5%) benefited from a disability insurance. Half of the relatives lived with PW-BD (52%), who were mainly suffering from bipolar type I disorder (71%). Partners, parents and siblings were represented in the same proportion (33.33%), with more female partners (5/7), more mothers (5/7) and more sisters (4/7). There were two drop-outs, a female partner who did not come to the appointment and finally did not wish to participate citing “poor timing,” and a mother who agreed to be included, but finally did not respond to the appointment.

### Overview of the Key Findings

#### Experiences of Stigma by Association (SBA)

There was no direct question on the interaction of families with the community, friends, and colleagues. These elements appeared during the interviews. Data from the transcripts showed that:

- Relatives’ experiences of coping with judgments or prejudices, or avoidant behaviors were mainly encountered in public settings (hospitalizations, in the streets, at work). SBA experiences could also happen in private settings (parties, lunch, and family reunion). They were reported as sneaky or open demeaning reactions from others. Pity was also experienced as a form of devaluation.
- These experiences aroused mainly when PW-BD was going through mania, hospitalizations, or hypomania. Thus, being too talkative, too uplifted, too arrogant, or exhibiting violent behavior, and also when being overly sedated by medication. When PW-BD was experiencing depression, devaluating comments were also experienced by relatives.
- Some relatives experienced judgment in their rural communities. Relatives living in the city did not describe experiences of coping in communities.
- Although the discriminative interactions took place between the relatives and doctors, or policemen, or colleagues, or friends, or the family (extended family, in-laws); these same categories of persons also provided support. Peer-support (in group or by meeting other persons/colleague related to person living with mental illness) was described as important, and distinct from other forms of support coming from persons with no similar direct experience.

SBA was not experienced by 30% of the sample. This group did not differ from the global sample on socio-demographic data, type of BD in PW, but it differed on the type of role as it was constituted of only fathers (two), and siblings (two brothers, and two sisters).

#### Role-Specific Data and Coping

Family members spontaneously reported that different prejudices were associated with the divergent family roles. Parents believed that they had to address responsibility, whereas partners had to consider the question of choice (separate or not from PW-BD). A mother (45–55) summarizes the parent versus partner dilemma: “I think that as parents we have an obligation towards our children, we can not abandon them… If we said to our child ‘this disease is so heavy, I do not want to take care of it any more, you're on your own,’ then I think this would really be stigmatized! it would be very hard to get people to accept it…it is much more difficult for parents with a bipolar child than for partners living with a bipolar person, because there is the possibility to separate, you can leave, there is not the same bond of absolute obligation that exists between parents and children...For a parent, no one says, ‘but why do you stay, why do you continue to take care of your children?’ It would be completely crazy. I believe stigma is higher for a partner than it is for parents.” The siblings who were interviewed were the ones who were implicated with their sibling living with bipolar disorder. They reported that the other siblings had little or no more contact with PW-BD. The relationship between the implicated sibling and PW-BD seemed both difficult and important. There was no culpability, but rather “a form of duty” (Sister, 50–60), and/or “… we have no obligation to be here, I choose to be here…” (Brother, 55–65).

Coping varied across the six different themes, and was also related to the roles. Parents geared their coping efforts towards acceptance: “Well, there's this and there's that and that could be a factor in the fact that he developed this (bipolar disorder), and there's a vulnerability. After facing that, I tell myself, ‘Okay, I'm his mother, but it's his life now.’ So I can not take everything and I'm letting go of that responsibility. He has his own life, I'm not the one who's going to lead his life for him, so that frees me up a bit” (Mother, 60–70). While partners had to position themselves in the dilemma of staying with someone they loved but with whom it was very difficult to live: “We say to ourselves ‘I'm fed up of living like this, but I love him, I'm staying anyway,’ the fact of staying leads us to start asking ourselves ‘But do I respect myself in fact?.’ Somebody else would leave in two seconds” (Female partner, 30–40). Siblings described acting as mediators between PW-BD and other members of the family or the community. “there is ‘the sick person,’ there is us and there are the others, we have to manage these three elements permanently, it can be exhausting because we never know how far the sick person can go and how far the others can tolerate and we are led to make this swinging movement between the two poles…” (Brother, 55–65). They were also asked to take accountability for their siblings living with bipolar disorder’s behavior: “the nicest ones will tell you: ‘Now you take your sick sister and you get the hell out of here’ (Brother, 55–65) or ‘you get your mad sister out of here’ (Sister, 50–60). These results are summarized in Table 1.

**Table 1:** Role related specificities

Role	Specific question	Specific answer
Parents	Responsibility	Acceptance
Partners	Choice	Position
Sibling	Duty	Mediator

#### Associative-Destigmatization Process (ADP)

**Fig. 1:**
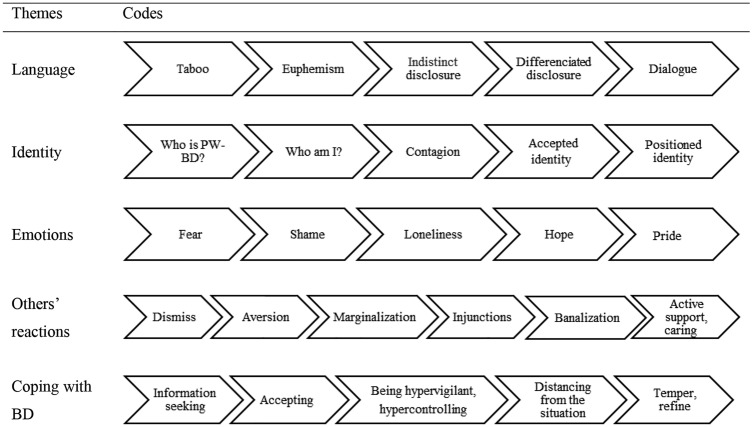
Associative-destigmatization process

The participants’ responses were grouped into codes that were then clustered into themes. There were five themes: “Language,” “Identity,” Emotions,” “Others’ reactions” and “Coping with BD”. These themes are constituted by either five or six codes, which represents a total of 26 codes. Associative-destigmatization process (ADP) is made from these five themes which codes can be organized in an ordered series that grows from difficulties to action (Fig. 1). ADP is a linear and progressive process.

**Fig. 2:**
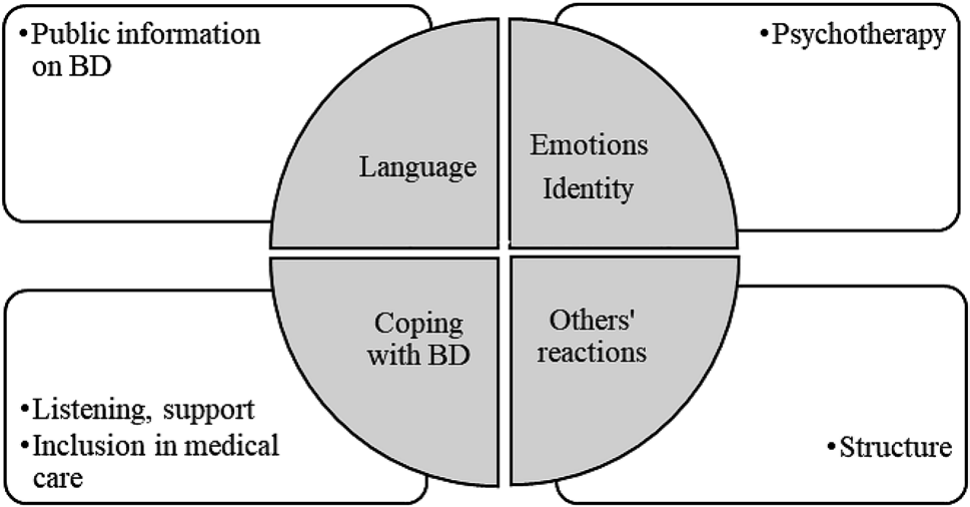
Interrelation between ADP and relative’s needs

One theme (“Relative’s Needs”) is constituted of independent codes (public information on BD, psychotherapy, Listening-support, inclusion in medical care, structure) that cannot be organized in a linear and progressive structure. Therefore it is not part of ADP, but Relative’s Needs elements register in response to the issues raised in ADP (Fig. 2).

##### Language

This theme was linked to disclosure, and reflected a process from “Taboo” to “Euphemism” to “Indistinct disclosure” to “Differentiated disclosure” leading to “Dialogue. “Taboo” referred to not naming PWL’s illness. The term “taboo” implies a restricted practice, and it seem appropriated to illustrate how relatives’ of PW-BD refrained from talking about the illness, even when making prior consent with their partner (PW-BD). It is taboo for the persons outside the intimacy of the couple or the family. “Euphemism” referred to when the relatives evoked BD in an incomplete or attenuated form, hiding the severity. “Indistinct Disclosure” indicated a rather awkward management of disclosure of the diagnosis. “Differentiated disclosure” referred to the conscious choice to disclose mental illness. Participants weighted the pros and cons of disclosure in a differentiated reasoning. As with “Taboo”, the decision was often made between the relative and the PW-BD. “Dialogue” referred to the fact that testifying about the illness allowed to open new relational fields.

##### Identity

This theme is linked to questioning and building identity of both the PW-BD and the relatives. It progresses through “Who is PW-BD?” to “Who am I?” to “Contagion” to “Accepted identity” and leads to “Positioned identity”. The code “Who is PW-BD?” referred to how relatives differentiate the person and the illness. Questioning the identity of the other leads to questioning one's own identity. The code “Who am I?” referred to the questioning about one’s identity due to his or her experiences of SBA. “Contagion” reflected how relatives used for the own experiences the same wording as those used to describe PW-BD”. “Accepted identity” expressed feelings of resignation and “Positioned identity” reflects how these experiences of dealing with SBA lead to personal growth.

##### Emotions

“Fear” reflected the anxiety when confronted with the illness and/or SBA. “Shame” referred to the experience of worthlessness in the context of SBA. The code “Loneliness” referred to moments of experienced separateness when confronted with the fact as to how the illness and/or stigma is affecting the family. The code “Hope” referred to a positive expectation for the future in coping with the illness. “Pride” expressed the identified accomplishments made by PW-BD and referred to expression of esteem for the PW-BDs’ courage in dealing with the situation.

##### Others’ Reactions

“Dismiss” reflected how others dismissed what was happening either by denial or withdrawal. “Aversion” included various aspects (judgment, jokes, insults, taking advantage of) that reflected feelings of dislike and/or disgust expressed by acquaintances, friends or extended family members of the relatives and PW-BD. The code “Marginalization” was coded when the relatives believed that they were excluded from social meetings. “Injunctions” refers to the experience of others telling the relatives what they should do. “Banalization” reflected how other persons did not understand the illness, which lead to stigma and “Active support, caring” reflected how others expressed acquired knowledge about the illness and displayed concern and kindness.

##### Coping with BD

The theme was linked to how the relatives adapted to the illness, using different strategies. The code “Information seeking” reflected how the relatives began by seeking information about the illness. “Accepting” referred to how the relatives assented to the reality of the situation. “Being hypervigilant, hypercontrolling” indicated how relatives acted with the PW-BD by overly checking what he or she was doing. “Distancing from the situation” reflected how the relatives detached from the situation, which helped to deal with what was happening. “Temper, refine” reflected how the relatives made minor but significant changes in their responses to the situation.

##### Relatives’ Needs

The theme “Relatives’ Needs” was the only subject constituted of independent elements. The participants spontaneously described aspects of what could be helpful for them on the social level (public information, structure), and on the personal level (psychotherapy, being listened to-receiving support). They also expressed the need of being included in PW-BDs’ medical care. “Public information” highlights the fact that relatives mentioned how public information about BD could help reduce stigma and SBA. “Structure” referred to the need of visiting places during periods of overwhelming distress in the relatives. “Psychotherapy” suggests that the relatives need personal psychotherapy to cope with the situation: “Being listened to, receiving support” indicate that the relatives need to talk about how they live or feel they are being listened to and given support. “Inclusion in medical care” reflected the need for some relatives to be taken into account in PW-BDs’ medical care.

The codes and themes are summarized in Table 2, and representative verbatim illustrate the categories. When first name were used by the participants, they were changed to ensure anonymization of the data. The interviewed relatives were categorized by their relational role with PW-BD and their age range. Both fathers were in the same age range, the three brothers were also in the same age range, some female partner also shared the same age range, as well as male partners and mothers.

**Table 2:** Summary of the data and findings

Themes	Codes	Verbatim (example)
Language	Taboo	“I use to hide it more” (mother, 55–60) “Clearly if it was cancer, we could tell it to everybody, This we can tell it to no one” (mother, 45–50)
	Euphemism	“I talk about it as if he had a cold.’” (father, 70–75) “First of all when I talk about it, I think I'm minimizing or I'm taking a kind of shortcut by saying that he has a problem” (mother, 55–60) “I said she had a serious neurological problem and the wrong medication and that's why she was hospitalized” (note: rather than saying it was mania that lead to the hospitalization) (mother, 45–50)
	Indistinct disclosure	“At first, I told a couple of friends that she likes a lot, I told them about it like this, I said "yes, she's bipolar, we just got the diagnosis" and she asked me: "why did you tell them?". And I said: «oops!» (female partner, 60–65) “I decided to talk about it right away as I had done for my epilepsy, but in fact, I think it would have been better if I had kept my mouth shut” (female partner, 30–35)
	Differentiated disclosure	“I think she has to decide who should know or not, and I ask permission when I think it's important for someone to know” (male partner, 35–45) “I realized that it really makes me feel good not to tell everyone about it” (sister, 45–50) “With close friends, family, it is not a problem, we don't talk about it, we are aware of that condition, there are no problems” (brother, 60–65)
	Dialogue	“One thing that I found positive was that when I disclosed it at work, one of my colleagues came to me and told me that he was going through it with his wife, the fact of talking about it uh allowed me to discuss it with someone and uh that I found out that it felt good…” (female partner, 40–45) “The discussion is totally open, the problems are known, there is no need to hide anything” (brother, 60–65)
Identity	Who is PW-BD?	“I would say they see that my wife is a good person; they said that she has a good heart…everybody says that it's strange she’s not like a normal person, she has days when she's very excited, she's always right; people they notice that” (male partner, 40–45) “It's very difficult to live with a bipolar person, because I very often find myself with a person I do not know at all (…). I also keep in my head the one I knew with her bursts of laughter (…) and I say to myself "it's her, it's always her" (female partner, 60–65) “She finds it even harder than I do to accept it because she tells to herself, "I do not know who my brother is anymore” (mother, 55–60)
	Who am I?	“… sometimes I am—I did not feel myself with him, i.e. sometimes I had to play a role to be not exactly what he wanted me to be but to not completely disagree with what he was saying” (female partner, 45–50) “I think that long before there were vulnerable spots in me, but living next to such ups and downs has weakened my identity” (female partner, 40–45)
	Contagion	“All of a sudden I came one day and asked him (her psychiatrist) do you think I'm bipolar?’ Because all of a sudden, I had a doubt, I asked myself, ‘am I bipolar?’” (mother, 60–65) “Well, for me, well, I think it's a bit of a decompensation in me too” (female partner, 35–40) “There are times when I have more energy, I have more desire to do things and then uh I do not know I do everything I want or I'm very positive, are there are different periods or do I also have the disease or what is it….” (male partner, 40–45)
	Accepted Identity	“Another impact of the illness is perhaps that she did not get married, we did not have grandchildren but that's how it is (…) I think you have to admit it” (father, 70–75) “Someone who has been through something, who is going through something and is dealing with it” (female partner, 40–45)
	Positioned Identity	“It's taught me that you can not keep it all inside, but I mean I have my own life and he has his own life, so uh, I'm not necessarily impacted” (sister, 50–55) “I am the mother of this person who has this problem” (mother, 55–60) “Telling myself that my family is made of eccentrics (…) and that it is part of my world and that I can be asserted in this context” (sister, 45–50)
Emotions	Fear	“So we were asking around, but we were not really going through with it, and I think it was out of fear, because we're afraid of what we're going to find out” (female partner, 50–55) “I confess that sometimes I am very scared, for myself, for those around me, for my partner” (sister, 45–50) “And I'll tell you that when I sleep with my sister in the same room, I'm not entirely reassured” (sister, 55–60)
	Shame	“I'd say I'm ashamed of my brother… I think it's a little close to shame, being ashamed of having a brother like that …” (brother, 60–65) “Shame is not there every day, but sometimes I felt shame; when she talked about personal things during a diner” (male partner, 40–45) “Of course we are ashamed, we do not want our friends to see our sister, we do not want our parents' friends to know that our daughter has a mental illness” (mother, 45–50)
	Loneliness	“I was under the impression that I was indeed being more isolated, and less understood” (sister, 25–30) “It's a vague sensation that I have but it's really very vague, I must say that I live with it very well…with a relative solitude yes loneliness” (mother, 70–75)
	Hope	“There’s always a hope that things will brighten; I think there's always hope: as long as there's life, there's hope” (brother, 60–65)
	Pride	“I think that if the patient has accepted the disease and is making sure that he takes care of himself and respects certain things that are important to maintain the balance, I would not even be afraid of being judged—I would be proud of it (inflection in the tone) because I've always admired people who are faced with very complicated situations and have the strength to laugh about it and even take advantage of the situation to do something about it” (female partner, 35–40) “I have a certain admiration for my daughters, they're not there yet, it's a long way to go, but they've really become aware of their limits, they have an ability to simply withdraw from social situations if they do not feel well” (mother, 45–50)
Others’ reactions	Dismiss	“My sister could not do anything about my daughter's hospitalization. In fact, she pulled out” (mother, 45–50) “I'm the only one in the family who really cares about her, who has contact, because my brothers have practically cut off all contact with her” (sister, 55–60) “Here people have no inclination to understand what is going on, they dismiss it” (female partner, 60–65)
	Aversion	“It may seem violent, they can find very hard words, yes very hard… almost insulting…(…) as ultimately some people will tell you: "well then you take your sick relative and you get the hell out of here or "you take your crazy relative out of here”, therefore they use the terms "your" sick person, "your" crazy person and thus it becomes your personal burden” (brother, 60–65) “Ah he married a bipolar or a crazy woman just to get a legal identity card” (male partner, 40–45)
	Marginalization	“It's because during the periods when she was really in difficulty or when she was hospitalized, all of a sudden there were not many friends who were present and we were much more alone as a family and even within the family…, people who do not understand at all and take their distance or are bluntly rejecting” (sister, 25–30) “There may be people who are going to be a little less close to us, who may be, I do not know, if say, if they have a party, they may not invite us, "rejection" yes we could be discarded because of the illness” (male partner, 40–45)
	Injunctions	“My sister tells me: ‘you have to send your daughter (PW-BD) to X (in another country), you just have to send her and then you cut all ties with her” (mother, 55–60) “A friend often told me that I was being too maternal with her» (sister, 55–60) “Sometimes people give you some pretty disturbing advice such as: “leave him, that’s enough”. People always say that it is a toxic relationship” (female partner, 40–45)
	Banalization	“…then if I try to explain to someone the disease he'll say: ‘well, it happens to me sometimes too, to drink and then I turn on the music’” (male partner, 40–45) “They do not understand. They say: « yeah well…it’s not serious”(mother, 55–60)
	Active support, caring	“When he is in a depressive phase, they (her children) are even touching, they will say let’s have a chat, let me take the dog out, do not worry; they are more helpful in daily life…” (female partner, 40–45) “Knowing that he (note: his director)understands, that he sympathizes with that and that every time I needed to stay home with her and not go to work, he understood” (male partner, 40–45) “The people I know are not going to express a strong opinion about what I should or should not do, they’re there when I need them, they’re there for good times, for bad times, but they’re not going to give me any advice or criticism about my life, they may just ask if they can do something for me” (female partner, 35–40)
Coping with BD	Information seeking	“I also did not know what «bipolar disorder» meant, and that’s when I started to search for information about it” (female partner, 35–40) “I’ve been learning for years, I’ve listened, I’ve asked around me, I’ve talked to doctors, I’ve read books, so I know what it is” (female partner, 35–40) “I’ve seen a few documentaries, but I do not remember very well when I understood what bipolarity is; I’ve read some things too” (female partner, 60–65)
	Accepting	“I think that working in the health field makes it a little bit easier to accept, not to take it as something scary and be more composed about it, but otherwise, I think it's still difficult to accept that fact (diagnosis)” (female partner, 40–45) “I could put a name to what was going on and I became less critical, I could understand what was going on and I would accept it better” (brother, 60–65)
	Being hypervigilant, hypercontrolling	“It's true that with Peter we are always a little bit anxious, every time we see him or when we do not have news for 2–3 days, the first question is "how are you doing?” (father, 70–75) “I pay attention to everything because I'm careful that it does not go too high or too low, therefore I'm always a bit unstable (…), I'm always a bit in control” (male partner, 40–45) “Knowledge about the illness is beneficial for the relationship and for the couple, but they may lead us to be hypervigilant” (female partner, 35–40)
	Distancing from the situation	“I feel concerned yes, responsible no, it's also attending the group for relatives that helped me a lot to step back” (sister, 25–30) “Okay I'm his mother but it's his life now. I can't take it all and I'm letting go of that responsibility. He's got his life, I'm not the one who's going to lead his life for him” (mother, 55–60) “I can not cover his illness, I can not solve all her inner worries I can't or do not want to anymore” (female partner, 40–45)
	Temper, refine	“For me it's the fine-tuning in daily life, it's delicate” (mother, 50–55) “It took some time to be able to empathize, to have empathy, but also enough distance to be able to protect and to be reassuring” (mother, 50–55)
Relatives’ needs	Public information	“For me it is important to inform my acquaintances about bipolarity because it's something that's still misunderstood by the general public. I mean to depict the main features (…), little booklets could be create such as: "What is bipolarity?" how to recognize it, how to recognize it in your child… to go into school pour inform students and teachers….” (mother, 45–50) “I think it's very important to inform people a little bit, so that they can understand, so that they can understand that it's an illness” (female partner, 60–65)
	Structure	“I became a member of an association of relatives of persons living with BD in France” (sister, 45–50) “I needed to rest, I just needed it and I had asked my doctor for a one month-treatment when I was particularly exhausted.” (sister, 45–50) “Do something for the family caregivers, give them a place they can go to, a place where they can recharge personal batteries, a place where they can sleep, a place where they can tell themselves:” Okay, now I can rest a little bit” (…); a place of their own where they can rest, recharge their batteries and then leave” (female partner, 60–65)
	Psychotherapy	“I'd never been worried about my mental health before and I've become worried lately, I had to start seeing someone (a psychotherapist), I had never imagined that for myself” (female partner, 50–55) “Now I have a psychiatrist and it has been good for me in other areas as well” (sister, 45–50) “We had a shrink for a long time, we also had couple therapy for a long time” (mother, 70–75)
	Being listened to, receiving support	“It's also nice to know that there are people who are there if you ever need to” (male partner, 40–45) “I think that the person who are close to a person with BD, that they also need to be listened to, probably much more than the patient himself, a lot of listening because they are the ones who are there with her everyday” (female partner, 40–45)
	Inclusion in medical care	“I found it really supportive that we are being taken into account, and that the health providers themselves are asking to talk to us, that there really is something that is being created between the family and the health care providers” (sister, 25–39) “I think there could have been a little bit of dialogue with the parents, because you're still in the front line of observing certain things” (mother, 50–55) “Collaboration with health care teams, not just a third person who is there just in case, but I think that there could be different ways to approach the partnership between families and friends, and the health care team and even with the institutions” (female partner, 35–40)

## Discussion

The aim of this qualitative research was to explore the stigma-related experiences of family members of PW-BD, and how relatives understand, experience and cope with SBA. To our knowledge, this is the first study to highlight specific issues and coping from family members related to their roles in the family.

### Stigma by Association

SBA was mainly experienced when during hypomanic, or mania was present in PW-BD, in the form of demeaning social interactions. Among the different codes in our study, “Shame” could be linked to disclosure and to aversion from others. Shamsaei et al. (2013) also showed that family members experienced stigma, shame, and social isolation. Corrigan and Miller (2004) pointed out that the relationship between shame and avoidance may be understood in terms of public stigma (persons would tend to avoid family members because of their association with people living with mental illness) or self-stigma (relatives would hide from the public). Different studies (Phelan et al. 1998; Osman and Kjellin 2002; Moses 2010; Mak and Cheung 2008) showed that about 25–50% family members endorsed being avoided by others or treated differently or being ostracized Relatives of our sample described being less invited by friends and/or relatives. They did not report avoiding others, but they choose as to whom they would like to disclose the diagnosis of their relatives. Most of them had discussed and agreed with PW-BD before making a disclosure. Disclosure of the relative's hospitalization seems to occur largely when unavoidable, as described by Phelan et al. (1998), and FM also could feel uncomfortable to disclose their household member's condition, as described by Chang and Horrocks (2006), and Koschorke et al. (2017). Perkins et al. (2018) reported that PW-MI and clinicians also use euphemistic labels due to fear of stigma or of damaging therapeutic relationships. Karnieli-Millers’ et al. (2013) findings suggest that families learn the “art of selective disclosure,” namely what, when, how much and with whom to share information. Pahwa et al. (2017) stated that the idea of disclosure is increasingly being linked to deliberate choices in PW-MI, especially since decisions about disclosure could have significant positive and negative repercussions (Greene et al. 2012). This was also described by the relatives of our study. Other studies (Bos et al. 2009; Chafetz and Barnes 1989) reported that openness about family members' MI can have positive consequences such as social support or help, and that it is likely to lessen stigmatizing responses. In our study, the last codes of the different themes are related to the experience of shared humanity.

Wisdom et al. (2008) analyzed identity-related themes in published self-narratives of people living with mental illness identity and their relatives. Writers gave an account of people living with mental illness identity as starting with a loss of self and identity, evolving to a duality (ill/well) selves, to perceptions of normality, to specific concerns about becoming a parent or not, and identity, and to hope and reconciliation. In our study, the duality expressed in the first code (“Who is PW-BD?”) related to differentiating the person and the illness. Contamination represents a subtle psychological process that results from associating with a person living with mental illness (Corrigan and Miller 2004). Children are especially likely to be perceived as contaminated by a parent’s mental illness. In our sample, “Contamination” was expressed by spouses rather than children, as they used to characterize their state of mind the terms used to describe mental illness.

The only theme constituted of non-progression elements (Relatives' needs) was instituted with needs expressed in the social dimension and needs expressed in the personal dimension. These elements give us an indication as to how to help relatives with different level interventions. This is particularly important, since stigma impacts treatment-seeking and participation in family groups that can be seen as both potentially supportive and threatening (Baron et al. 2019).

The qualitative data showed that ADP is not a static but a dynamic process, which gradually evolves in five distinct dimensions: language, identity, emotions, others’ reactions and coping with bipolar disorder. ADP is a two factor entity that distinguishes content (steps) and process (structure) among relatives of PW-BD. ADP can be compared to the recovery process, as both describe a growth from dealing with difficulties to taking action through identified steps.

Six relatives reported no personal experience of SBA. This indifference to stigma has been described by Corrigan and Watson (2002) in people living with mental illness, but to date it has not been described in their relatives. Moreover, in this sample the relatives did not differ from the global sample on sociodemographic, but rather on the specificity of family roles (fathers and siblings). We did not find similar data in the literature. Further research is needed to clarify the characteristics associated with these responses.

### Coping with Roles

In our study, parents, partners and siblings indicated that they have to deal with specific dilemma, and family memebers said spontaneously that their experiences of SBA were mediated by their family roles. Parents talked about responsibility. Zhang et al. (2018) found that parents of person living with mental illness felt stigmatized and had difficulties coping with SBA. Research has suggested that the public perceives family members, especially parents, as responsible for the relative's mental illness (Corrigan and Miller 2004). In our sample, mothers questioned the type of education they had given, while fathers were implicated in their child’s social integration (such as work). This gendered difference has also been described by van der Sanden et al. (2015), and in an analysis of a television series by Smith-Frigerio (2018). Mothers are depicted as primary caretakers, and as being overprotective, while fathers are portrayed as less talkative. Chang et al. (2017) showed that parents had a higher SBA than spouses did. Concerns regarding diminished status was expressed more by wives of men admitted to a state psychiatric hospital, than by parents (Ostman and Kjellin 2002). In contrast to biological relatives, partners are seen as having chosen to affiliate with someone with MI. In our sample, this question of choice and its impact were spontaneously discussed by the partners of PW-BD, because their friends or relatives were questioning them about it. From data of a non-experimental study, Perel (2017) reported that partners who had been cheated were ashamed when choosing to stay in the relationship. She stated that while divorce was once associated with stigma, nowadays choosing to stay after an infidelity is the new shame. Although it was not reported in our sample, theoretically, partners of PW-BD could be caught in a double stigma if PW-BD have affairs during mania. As far as siblings are concerned, it is considered their duty to help person living with mental illness with the necessary treatment adherence to avoid relapses (Corrigan and Miller 2004). The siblings who participated in our study did not mention being concerned with the management of the illness, but reported being held as responsible for their relatives' behavior, and they reported acting as mediators between PW-BD and other persons in their surroundings.

### Interventions

Family members spontaneously expressed different features that could help them cope with bipolar disorder in the family and the impact of stigma (public information, structure, psychotherapy, listening to/receiving support and being included in PW-BD medical care). Our results also showed that SBA is specific to each family role, and that siblings who remain relatively invisible in clinical service as well as in research studies (Sin et al. 2015) play an important part as mediators between PW-BD and other members of the family or the community. Family members should have access to social, informational, instrumental and emotional support provided by community members and mental health professionals (Sveinbjarnardottir and Dierckx de Casterle 1997; van der Sanden et al. 2014, 2015), and interventions that target individual- and family-level stigma-related feelings of embarrassment (Ahmedani et al. 2013). In a meta-analysis, Shi et al. (2019) noted that the caregivers of person living with mental illness might benefit from further support (e.g. psychoeducation) to improve their knowledge about such illnesses. Corrigan and Nieweglowski (2019) hypothesized a U-shaped relationship between familiarity and stigma (greater familiarity lead to less public stigma), and asserted that in Western Europe, nuclear family members (parents, siblings, spouse, children) are more familiar with person living with mental illness than relatives of the extended family, co-workers or friends. The U-shaped curve suggests anti-stigma programs may need to differentially address the inverse relationship between familiarity/public stigma and the positive relationship, and that contact-based interventions need to communicate the essential humanity of person living with mental illness, rather than knowledge. Schlier and Lincoln (2019) tested how implicit categorization based on mental health status were used to organize information. When two mental illness groups were presented together (schizophrenia and depression), there was no use of specific diagnostic categories. The general population considered broadly “people with mental illness” versus “people without mental illness.” Mapping implicit categorization and stigmatization could inform anti-stigma interventions. Based on «Relative’s needs» theme, favored intervention was twofold. First, the development of campaigns to enhance programs and education to improve public’s knowledge regarding bipolar disorder and the impact of the illness on PW-BD and on the relatives or family members. These information could help diminish public prejudice and ease group cooperation and social engagement. Second, the development of programs for relatives’ of PW-BD to help discuss SBA and its impact on family members and in their different roles. In this sample, the family roles determined specific questions, and therefore different coping strategies. Further studies are needed to explore the role-specific features of the relatives’ of PW-BD.

### Limitations

This study has several limitations. First, our study focused on relatives from the immediate household in different roles (parents, partners and siblings). Children were not recruited since we had limited access to them. Moreover, previous studies have already assessed SBA among children of parents with serious mental illness (Bee et al. 2014). Secondly, the study recruited a purposive sample with the size determined by data saturation, with the expectation that each participant would provide unique and valuable information for the study. This may limit the generalizability of the findings (Etikan et al. 2016; Valerio et al. 2016).

## Conclusions

Qualitative studies provide an in-depth understanding of lived human experiences in the context in which they occur. Family members reported experiences and coping with SBA in five different dimensions. The SBA experiences were also related to specific family roles. These results provide landmarks for targeted interventions for relatives of PW-BD. Further studies are needed to validate this process and further explore its clinical and theoretical implications. Further knowledge could be enhanced by peer family researchers’ collaboration, who may provide advice in the design of the study, and also elicit more features in the data collection. Mixed-methods data could also bring additional insight in the description SBA and its’ interaction with ADP.
